# Long-term effectiveness and safety of self-management of oral anticoagulants in real-world settings

**DOI:** 10.1186/s12872-019-1168-2

**Published:** 2019-08-02

**Authors:** Bárbara Menéndez-Jándula, José Antonio García-Erce, Clara Zazo, Luis Larrad-Mur

**Affiliations:** 1 Hospital San Jorge, Av. Martínez de Velasco 36, 22004 Huesca, Spain; 2 Banco de Sangre y Tejidos de Navarra, Pamplona, Spain; 3 Department of Medicine, University of Zaragoza, Zaragoza, Spain

**Keywords:** Anticoagulants, Oral, Self-management, Coagulometer, Effectiveness, Safety

## Abstract

**Background:**

The patient self-management (PSM) is an effective approach for controlling the international normalized ratio, INR, within the therapeutic range. Nevertheless, most of the literature derives from randomized clinical trials, and no from routine clinical practice. The main objective of the present study was to evaluate long-term effectiveness and safety of PSM of oral anticoagulants (OACs) in real-world settings.

**Methods:**

This prospective cohort study involved 808 patients who were trained for PSM between July 2009 and March 2012, and followed-up for a maximum observational period of 5 years. The follow-up consisted of a visit to the physician every 6 months. All patients used the same type of portable coagulometer, able to store digitally up to 100 INR measurements. Effectiveness outcomes included the percentage of patients within the therapeutic range, the time within therapeutic range (TTR), and the evolution of the TTR over 365 days of follow-up. Long-term safety profile of PSM included the incidence of all-cause deaths and complications (thromboembolic or hemorrhagic) reported between July 2009 and June 2014, and the time to event.

**Results:**

The median follow-up was 3.3 years. The percentage of patients within therapeutic INR target range was 67.5%. The median TTR was 71.5%. The TTR increased over the follow-up period, either overall and regarding target INR*.* All-cause mortality was 2.4 per 100 patient-years (59 cases). The thromboembolic event rate was 0.9 per 100 patient-years (24 cases). The rate of major hemorrhages was 0.45 per 100 patient-years. Patients who drop out the PSM to perform the conventional management had greater rates of complications: 2.4, 1.8, and 3.4 per 100 patient-years for thromboembolic complications, major hemorrhagic events, and mortality, respectively.

**Conclusions:**

The PSM of OACs is effective for maintaining patients within the INR therapeutic range for a long period of time in routine clinical practice. Results of the present study suggest that its effectiveness is at least comparable to the conventional management. Moreover, it seems safe in real-world settings, by preventing all-cause mortality, and thromboembolic and major hemorrhagic complications.

**Trial registration:**

This study was not a trial, thus registration was not required.

## Background

Oral anticoagulants (OACs) are a group of medicines indicated for preventing thromboembolic events in susceptible patients [1]. One of the limitations associated with conventional OACs, such as acenocoumarol (the most prescribed in Spain), is their pharmacokinetic variability [2]. Patients, frequently aged over 65 years, are required to periodically visit healthcare centers for routine monitoring (checking out if the international normalized ratio, INR, is within the therapeutic range) and dose adjustments [3, 4]. The INR therapeutic range includes values associated with the lowest risk for experiencing thromboembolic or hemorrhagic events [5]. Patient self-management (PSM) of OACs, i.e. self-testing in combination with self-adjustment of doses, has emerged as an alternative to the conventional management, especially after the development of coagulometers [6, 7]. Nowadays, portable coagulometers are effective tools for monitoring INR in an easy and reliable way [8]. By performing PSM, the patient avoids periodic visits to the healthcare center. In fact, it has been associated with an improvement in the quality of life [9]. International guidelines recommend performing PSM for long-term conditions, established the target INR, and having been trained by a healthcare provider [10, 11]. Some studies have reported a higher time within the INR therapeutic range in patients performing PSM, compared with the conventional management [12, 13]. Furthermore, PSM of OACs have also been associated with a significant reduction in the number of treatment complications [14]. There exists enough clinical evidence to confirm that PSM is an effective approach for controlling INR within the therapeutic range in patients receiving OACs. Nevertheless, most of the literature derives from randomized clinical trials or short-term prospective studies [13, 14]. Therefore, the main objective of the present study was to evaluate long-term effectiveness and safety of PSM of OACs in real-world settings.

## Methods

### Study design and data source

Between July 2009 and March 2012, the regional Government of Aragon (Spain) conducted a project for improving the accessibility of patients to the management of OAC. Physician of primary and specialized centers from the region, could offer, to those patients that they considered appropriate, perform PSM of OAC. This prospective cohort study involved the patients who agreed to PSM and who received training between July 2009 and March 2012. Physicians could recruit to PSM (inclusion criteria): adults or legal minors (with a responsible caregiver); with no physical or mental impairment (or with a responsible caregiver); requiring long-term OAC treatment (> 3 months), and willing to provide data from the coagulometer. The study consisted of the following periods: recruitment, training of healthcare professionals, training of patients (initiated in July 2009), and follow-up of patients (from the end of training to June 31st 2014). A training course was offered to all healthcare providers who were interested in learning about PSM, and consisted of 3 sessions (1 theoretical and 2 practical). Patients also received a course, consisting of a theorical and a practical session. In the practical session, patients learned to use the coagulometer, collect the blood sample, and adjust the dose. All patients used the same type of portable coagulometer (CoaguChek® XS, Roche Diagnostics), able to store digitally up to 100 INR measurements. The follow-up consisted of a visit to the physician every 6 months aimed at: downloading INR measurements from the coagulometer (by using Tao Net, Roche Diagnostic); reporting treatment-related complications; and verifying the dosing. All prospective data were subsequently collected by the main investigators for analysis. Demographic and clinical information from patients was provided from medical records. All patients signed the written informed consent to participate in the study. Procedures were in concordance with the Declaration of Helsinki, and the Ethical Committee of Aragon.

### Study variables

The effectiveness of PSM of OACs was determined by using INR measurements that were stored in the coagulometer. Only patients who downloaded the INR measurements at least one time from their coagulometers were included in the effectiveness analysis. The PSM was considered effective if INR was within therapeutic target range. Effectiveness outcomes included the percentage of patients within the therapeutic range, the time within therapeutic range (TTR), and the evolution of the TTR over 365 days of follow-up. Baseline, day 0, or study initiation was the date in which the patient got the coagulometer and started to PSM. Long-term safety profile of PSM included the incidence of all-cause deaths and complications (thromboembolic or hemorrhagic) reported between July 2009 and June 2014, and the time to event (TTE). The TTE was calculated as the elapsed time between the initiation of the patient to PSM and the development of the event. Hemorrhagic complications were classified as major or minor, in accordance with the criteria of the International Society of Thrombosis and Haemostasis [15].

### Statistical analyses

Continuous variables were expressed as the mean, median, standard deviation (SD), or interquartile range (IQR, i.e. first and third quartile of the distribution of values), whereas categorical ones as absolute and relative frequencies. The Rosendaal linear interpolation method was used to calculate the TTR in each patient [16]. The correlation between the percentage of patients within therapeutic INR target range and TTR was analyzed by using Spearman’s correlation test. Differences in TTR regarding demographic and clinical characteristics of patients were performed with the T, Mann-Whitney o Kruskal-Wallis tests, when appropriate. The evolution of TTR (after 30, 90, 180, and 365 days since study initiation) was analyzed by using the paired-T test. Comparisons of TTR at different time points were carried out by using the one-way ANOVA and Tukey’s HSD post hoc tests. The impact of demographic and clinical characteristics of patients on safety variables was evaluated by using the independent samples T test. Survival estimations were calculated by using the Kaplan-Meier methodology and Cox regression analyses (Hazard ratio, HR, 95% confidence interval, 95% CI). Covariates evaluated in these analyses were: follow-up period (≤2 years versus > 2 years), TTR (≤65% versus > 65%), and age (≤60 years versus > 60). Safety variables depending on the time of follow-up (first 2 years versus after 2 years) were evaluated by using the Fisher Exact test. Logistic regression models were also created to identify variables associated with experiencing an event (mortality or any complication). Variables initially included in modes were: age, gender, indication for anticoagulation, target INR, and efficacy outcomes (TTR, TTE). All demographic and clinical variables from patients were included in the model (Odd ratio, OR). The incidence of events (death, and thromboembolic or hemorrhagic complications) were finally compared between patients who performed PSM during the follow-up period and those who did not so. Statistical significance was established when *P* ≤ 0.05. All statistical procedures were performed with SPSS 15.0 version.

## Results

### Patients

**Fig. 1:**
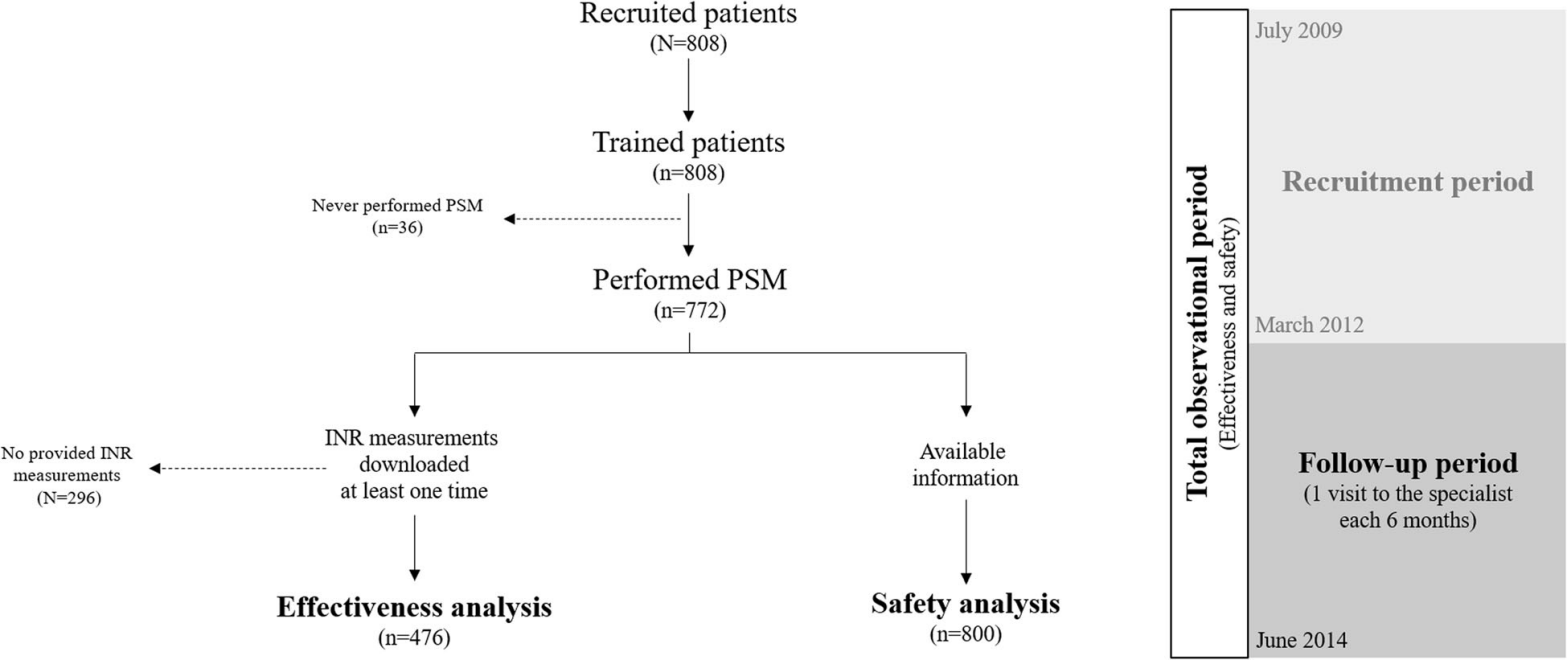
Flowchart of patients and phases of the study. PSM, patient self-management; INR, international normalized ratio

A total of 808 patients were included in the study (Fig. 1). Demographic and clinical characteristics of patients and treatments are shown in Table 1. Patients were predominantly male (61.8%), aged between 60 and 75 years (38.5%), receiving acenocoumarol (97.9%), with a target INR between 2.0 and 3.0 (75.5%), for preventing mainly atrial fibrillation/atrial flutter (42.6%). The median follow-up period was 3.3 years (IQR 2.4–4.0). A total of 107 patients (14.0%) required a responsible caregiver to perform adequately PSM. At the end of the study, 631 patients (78.1%) continued with PSM. Death (7.3%) and end of treatment (5.2%) were the main reasons of study withdrawal.

**Table 1:** Demographic and clinical characteristics of patients and treatments

	Patients (*N* = 808)
Gender, n (%)	
Male	499 (61.8)
Female	309 (38.2)
Age groups, n (%)	
< 50	168 (20.8)
50–60	197 (24.4)
60–75	311 (38.5)
> 75	132 (16.3)
Origin of the patient, n (%)	
Primary healthcare	340 (42.1)
Specialized healthcare	468 (57.9)
Anticoagulant agent, n (%)	
Acenocoumarol	791 (97.9)
Warfarin	17 (2.1)
Target INR, n (%)	
2.0–3.0	610 (75.5)
2.5–3.5	182 (22.5)
Other	16 (2.0)
Indication for anticoagulation, n (%)	
Mechanical heart valve	204 (25.2)
Atrial fibrillation/ Atrial flutter	344 (42.6)
Venous and arterial thrombosis	111 (13.7)
Others	148 (18.3)
Unknown	1 (0.1)
Time of follow-up, median years (IQR)	3.3 (0.0–5.0)
Required a responsible caregiver to PSM ^a^	107 (14.0)
Study withdrawal, n (%)	177 (21.9)
Death	59 (7.3)
End of treatment	42 (5.2)
Never performed PSM	36 (4.5)
Voluntary withdrawal	28 (3.5)
Loss to follow-up	8 (1.0)
Others	4 (0.5)

*INR* international normalized ratio, *IQR* interquartile range, *PSM* patient self-management

^a^ Data were not available in 42 patients

### Effectiveness of PSM

**Fig. 2:**
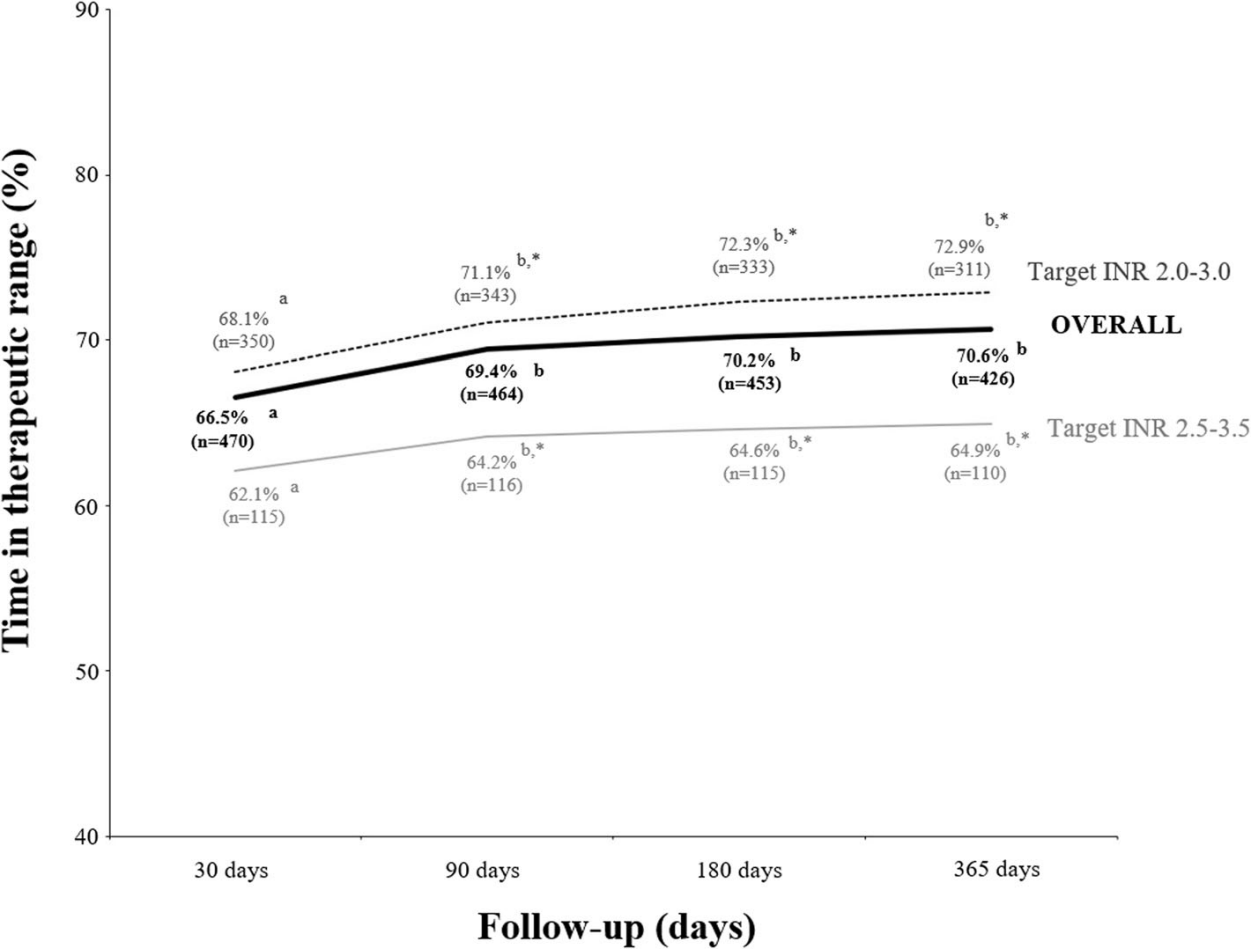
Evolution of the time in therapeutic range regarding target international normalized ratio. Different letters (^a, b^) indicate significant differences (*P* < 0.01) between time points, whereas asterisks do between groups (2.0–3.0 versus 2.5–3.5)

Of 808 patients, 476 (58.9%) downloaded the INR measurements at least one time from their coagulometers; therefore, they were included in the effectiveness analysis. The percentage of patients within therapeutic INR target range was 67.5% (from 49,982 INR measurements, in a total of 385,573 days of follow-up). The median TTR was 71.5%. The TTR was below 60% in 81 patients (17.0%), between 60 and 70% in 130 (27.3%), and above 70% in 265 patients (55.7%). A significant positive linear correlation was found between the percentage of patients within therapeutic INR target range and the TTR (Spearman’s rho correlation coefficient: 0.92; *P* < 0.001). Time in therapeutic range regarding demographic and clinical characteristics of patients is shown in Table 2. Significant differences in TTR was found regarding: gender (higher in women, 72.6%, versus men, 70.6%; *P* < 0.026), target INR (higher in INR 2.0–3.0, 73.9%, versus INR 2.5–3.5, 65.6%; *P* < 0.001), and indication for anticoagulation (higher in atrial fibrillation/flutter, 73.5%, and venous and arterial thrombosis, 73.1%, versus mechanical heart valve, 67.2%; P < 0.001). No differences in TTR were found regarding age group, and origin of the patient (data not shown). The TTR increased over the follow-up period, both overall and regarding target INR (Fig. 2). However, significance differences (*P* < 0.05) were only found between 30 days (after study start) and the following time points (90, 180, and 365 days). Patients with target INR between 2.0 and 3.0 showed significant higher TTR after 90 (71.1%), 180 (72.3%), and 365 days (72.9%) than those between 2.5 and 3.5 (64.2, 64.6, and 64.9%, respectively, *P* < 0.01).

**Table 2:** Time in therapeutic range regarding demographic and clinical characteristics of patients

	Median time in therapeutic range (%)	*P*
Overall (*n* = 476) ^a^	71.5	
Gender		< 0.026
Male	70.6	
Female	72.6	
Target INR		< 0.001
2.0–3.0	73.9	
2.5–3.5	65.6	
Other	67.6	
Indication for anticoagulation		< 0.001
Mechanical heart valve (*n* = 125)	67.2	
Atrial fibrillation/ Atrial flutter (*n* = 178)	73.5	
Venous and arterial thrombosis (*n* = 57)	73.1	
Others (*n* = 93)	71.7	

^a^ Patients included in the effectiveness analysis

### Safety of PSM

**Fig. 3:**
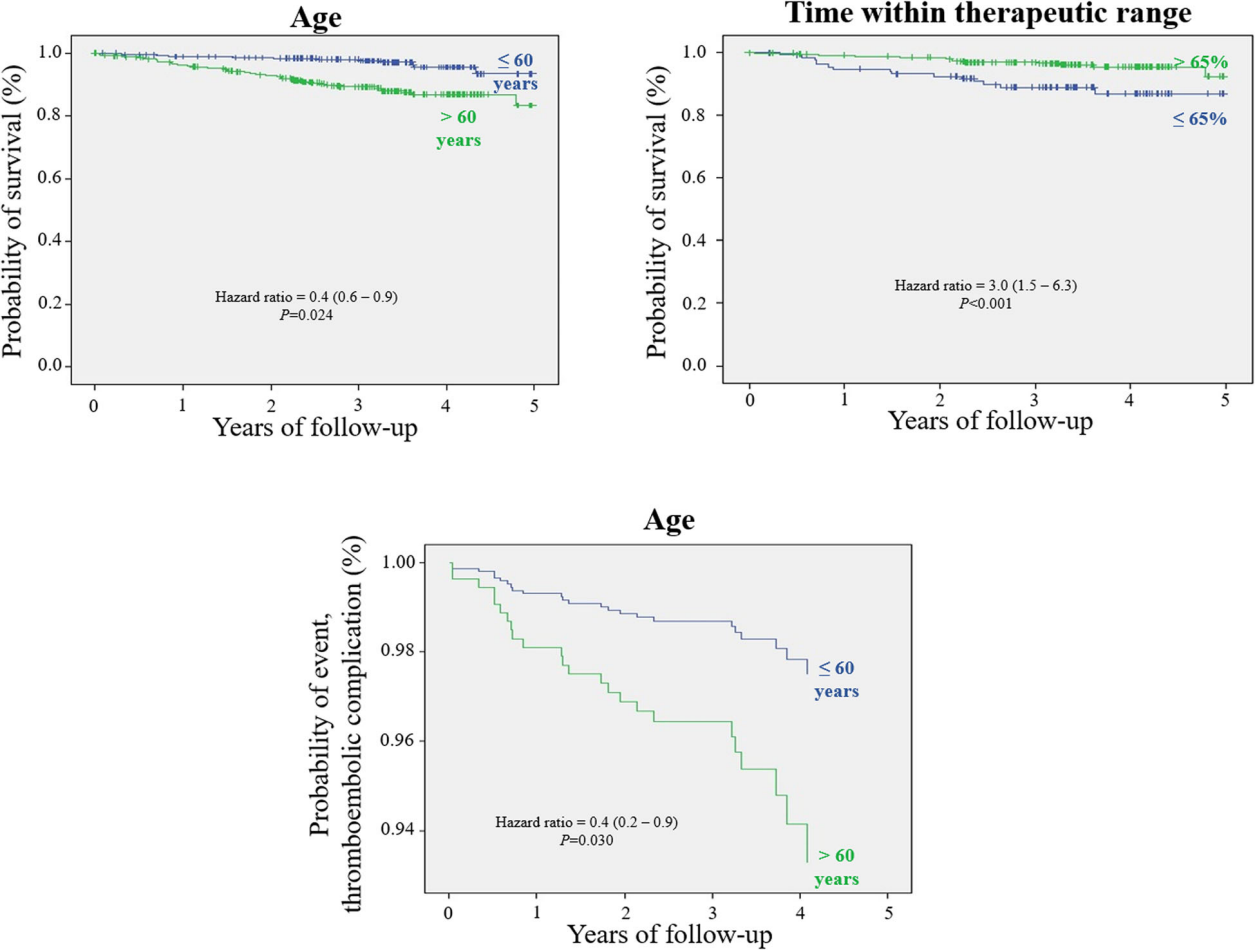
Survival curves depending on age and time within therapeutic range, and probability of thromboembolic complication depending on age

Of 808 patients, information about safety was available in 800 (99.0%); thus, they were included in the safety analysis. Fifty-nine patients died during the study period. All-cause mortality rate was 2.4 per 100 patient-years. The mean age of patients who died was significantly higher (71.8 years, SD 11.8) than those who did not so (61.0 years, SD 13.6). The incidence of mortality was higher among patients with atrial fibrillation (3.1 per 100 patient-years) compared with other indications, although the difference was not statistically significant. The mean TTR of patients who died was significantly lower (63.3%, SD 12.0) than who did not so (70.4%, SD 12.5, *P* < 0.03). The risk of mortality was significantly different depending on the following factors: follow-up period (≤ 2 years versus > 2 years; OR 17.1, 95% CI 7.1–40.8; *P* = 0.001), and TTR (≤ 65% versus > 65%; OR 2.3, 95% CI 1.5–4.0; *P* = 0.049;). Cox regression models (for survival) were significantly different depending on: age (≤ 60 years vs > 60 years, HR 0.4, 95% CI 0.6–0.9; *P* = 0.024), and TTR (≤ 65% versus > 65%; HR 3.0, 95% CI 1.5–6.3; *P* < 0.001 (Fig. 3). Of 59 patients who died, 34 (57.6%) did during first 2 years of follow-up. A total of 24 venous and arterial thromboembolic complications (especially stroke, *n* = 20) were reported during the follow-up period. Its incidence rate was 0.9 per 100 patient-years. The mean TTE was 20.6 months (SD 16.4). Three of the complications were fatal: a pulmonary thromboembolism and two cerebrovascular accidents. The mean age of patients who suffered a thromboembolic complication was significantly higher (68.8 years, SD 11.4) than those who did not so (61.1 years, SD 13.8, *P* < 0.006). Cox regression models (for thromboembolic complication) were significantly different depending on age (≤ 60 years versus > 60, HR 0.4 95%CI 0.15–0.92; *P* = 0.030). No significant differences in the incidence of thromboembolic complications were found regarding TTR or target INR. A total of 117 hemorrhagic complications (11 of them major) were reported during the study period. The incidence rate of major hemorrhages was 0.5 per 100 patient-years. The TTE (severe) was 18.9 months (SD 17.3). The mean TTE was significantly lower for women than men (6.6 versus 22.3 months, *P* < 0.001). Gastrointestinal (*n* = 5) was the most frequent major hemorrhagic complication. None of hemorrhagic complications resulted in death. During the first 2 years of follow-up occurred significantly more cases of death (34 cases, 57.6%), and thromboembolic (16 cases, 66.7%) and major hemorrhagic complications (7 cases, 63.6%) than after 2 years (*P* < 0.001 in all cases). The mean age of patients who experienced any event (death or any complication; analyzed together, *n* = 82) was significantly higher (70.2, SD 11.3) than those who did not so (60.8, SD 13.7). The risk of experiencing an event was significantly higher in patients who did not required a responsible caregiver than who did so (OR 0.19, 95% CI 0.1–0.3; *P* < 0.001). The remaining demographic and clinical characteristics of patients showed no significant correlations with experiencing an event. Patients who performed the conventional management, i.e. received training for PSM but never performed it (*n* = 36), or who stopped PSM (*n* = 16) or continued with routine monitoring (*n* = 68), had greater incidence rates of complications: 2.4, 1.8, and 3.4 per 100 patient-years for thromboembolic complications, major hemorrhagic events, and mortality, respectively. The mean TTE was 29.4 months, 15.5 months and 20.8 months.

## Discussion

During more than six decades, OACs with vitamin K antagonists have been the standard treatment for preventing thromboembolic events [17]. Nevertheless, periodic visits to the healthcare center for monitoring became a real problem for patients, especially those with advanced age. The PSM has emerged, in part, with the aim of solving such a limitation [5–7]. Results from our study demonstrate the effectiveness and safety of PSM for long-term periods of time in real-world settings. To date, the evidence of PSM, demonstrating the improvement in the quality of anticoagulation and the reduction in complications, derives mainly from randomized clinical studies (with strict inclusion and exclusion criteria), and short-term periods of follow-up. Henegan et al., [14] in a systematic review and meta-analysis, involving 11 randomized trials and 6417 patients using PSM (self-testing and self-dosage), demonstrated a significant reduction in thromboembolic complications (Odd ratio, OR 0.51) but not in hemorrhagic ones or deaths. Bloomfield et al., [18] in a meta-analysis of 22 studies with 8413 patients assigned to self-testing or PSM, revealed significant reduction in thromboembolic complications (OR 0.58) and deaths (OR 0.74). The time in therapeutic range has also been proven to be longer in PSM (ranging between 61.3 and 83.0% after 12 months of follow-up) than using the conventional management (61.0–70.8%) [19–22]. Menéndez-Jándula et al., [23] in a randomized trial compared the efficacy and safety of PSM of OACs and the conventional management in 737 Spanish patients for a median of 11.8 months. No differences were found between TTR using PSM (64.3%) and the conventional management (64.9%). The number of major complications was higher in patients from the conventional management (27) than PSM (8). In our knowledge, limited studies have been specifically designed to long-term evaluate effectiveness and safety of PSM in real-world settings [24–28]. The summary of main studies involving PSM in real-world settings are shown in Table 3. Fritschi et al., [24] in a study with 330 patients performing PSM in Switzerland, reported a TTR of 72.0% after 2.8 years of follow-up. The incidence rate of thromboembolic and major hemorrhagic events was the same, 0.6 per 100 patient-years. Matchar et al., [25] with 1463 patients performing PSM, showed a TTR of 66.2%. Incidence rates of mortality and major hemorrhages were 3.4 and 4.0 per 100 patient-years. Nagler et al., [26] involving 1221 patients trained for PSM and followed-up for a median of 4.3 years, showed a TTR of 80%. The incidence rate of mortality, and thromboembolic events, and hemorrhagic were 1.4, 0.9, and 1.2 per 100 patient-years, respectively. Grove et al., [28] in a recent study aimed at comparing PSM of OACs with direct OACs in patients with atrial fibrillation revealed an annual incidence rate of 1.1 for mortality, 0.5 for thromboembolic events, and 2.3 for major hemorrhages. Results from our present study are in concordance with literature, regarding TTR and incidence rates of events. Significant differences in TTR between males and females have been previously reported by other authors [29]; however, differences in TTR after 90 days since study start have not been so. In our knowledge, there is not an easy explanation for such observation. The mean TTE for patients in PSM was around 20 months, and the highest proportion of events occurred in the first 2 years, in contrast with those who stopped PSM for any reason. The incidence of complications and mortality was much higher in patients who stopped performing PSM. In the case of thromboembolic and hemorrhagic complications, the incidence rate was more than two-fold.

**Table 3:** Summary of main studies involving patient self-management of oral anticoagulants in real-world settings

Author	Number of patients	Indication	Median Years of follow-up	Mortality ^a^	Thromboembolic events ^a^	Major hemorrhages ^a^	Median TTR (%)
Fritschi et al., [24]	300	Mixed	2.8	–	0.6	0.6	72.0
Matchar et al., [25]	1463	AF, MHV	3.0 ^b^	3.4	0.7	4.0	66.2
Nagler et al., [26]	1221	Mixed	4.3	1.4	0.9	1.2	80.0
Ward et al., [27]	296	Mixed	1.0	–	–	–	75.3 ^**b**^
Grove et al., [28]	534	AF	2.5	1.1	0.5	2.3	–
Present study	808	Mixed	3.3	2.4	0.9	0.5	71.5

*TTR* time within therapeutic range, *AF* atrial fibrillation, *MHV* mechanical heart valve

^**a**^ Incidences rates (per 100 patient-years); ^**b**^ Values are mean

One of the limitations of the study was the absence of a proper control group, for comparison purposes. Although a control group would have improved the design of the study, we aimed at carrying out a study in real-world conditions, and a large number of patients. Beside this, and cautiously, the study also provides information about patients who did not perform the PSM and continued in the conventional management (in low number, due to the same nature of real-world settings). Furthermore, comparisons were possible by using the published national and international literature. Another limitation was the lack of some effectiveness data (only 58.9% of patients downloaded the INR measurements). However, this limitation was intrinsically associated to observational, clinical practice studies.

## Conclusions

The PSM of OACs is effective for maintaining patients within the INR therapeutic range for a long period of time in routine clinical practice. Results of the present study suggest that its effectiveness is at least comparable to the conventional management. Moreover, it seems safe in real-world settings, by preventing all-cause mortality, and thromboembolic and major hemorrhagic complications. Additional studies in clinical practice, involving control groups and a larger cohort of patients are required to corroborate these results.

## Data Availability

The datasets used and/or analysed during the current study are available from the corresponding author on reasonable request.

## Abbreviations

CI: Confidence interval
HR: Hazard ratio
INR: International normalized ratio
IQR: Interquartile range
OAC: Oral anticoagulant
PSM: Patient self-management
SD: Standard deviation
TTR: Time within therapeutic range
