# Correction to: Long-term effectiveness and safety of self-management of oral anticoagulants in real-world settings

**DOI:** 10.1186/s12872-019-1268-z

**Published:** 2019-11-26

**Authors:** Bárbara Menéndez-Jándula, José Antonio García-Erce, Clara Zazo, Luis Larrad-Mur

**Affiliations:** 1 Hospital San Jorge, Av. Martínez de Velasco 36, 22004 Huesca, Spain; 2 Bancode Sangre y Tejidos de Navarra, Pamplona, Spain; 3 Department of Medicine, University of Zaragoza, Zaragoza, Spain

**Correction to: BMC Cardiovasc Disord (2019) 19:186**

**https://doi.org/10.1186/s12872-019-1168-2**

After publication of the original article [1], we were notified that the name of the software mentioned in the Background section is TAONET and not Tao Net.

## References

[1] Menéndez-Jándula (2019). BMC Cardiovasc Disord.

